# LY6H is a marker of human pancreatic delta cells

**DOI:** 10.1038/s41598-025-18321-2

**Published:** 2025-09-26

**Authors:** Jacqueline V. Schiesser, Yi Yu, Thomas Loudovaris, Helen E. Thomas, Andrew G. Elefanty, Edouard G. Stanley

**Affiliations:** 1 Murdoch Children’s Research Institute, The Royal Children’s Hospital, Flemington Road, Parkville, VIC 3052 Australia; 2 The Novo Nordisk Center for Stem Cell Medicine (reNEW), Murdoch Children’s Research Institute, Parkville, VIC 3052 Australia; 3 Department of Paediatrics, Faculty of Medicine, Dentistry and Health Sciences, University of Melbourne, Parkville, VIC 3052 Australia; 4 St. Vincent’s Institute, Fitzroy, VIC 3065 Australia; 5 Department of Medicine, St. Vincent’s Hospital, University of Melbourne, Fitzroy, VIC 3065 Australia

**Keywords:** Delta cell, Type 1 diabetes, Cell surface markers, Islet, Cell isolation, Gene expression analysis, Diabetes

## Abstract

The identification of cell surface markers specific to pancreatic islet cell subsets is important for both the study of islet biology and for investigating the pathophysiology of diseases in which these cell types are lost or damaged. Analysis of publicly available single-cell RNAseq data showed that *LY6H* transcripts are highly enriched in the delta cells of the pancreas. This finding was confirmed using immunofluorescence analysis of histological sections of human pancreas, and flow cytometric analysis of human islet preparations. We found that expression of LY6H was robustly associated with pancreatic delta cells in samples derived from both control and diabetic donors. Furthermore, we demonstrate that antibodies against LY6H can be used to substantially enrich for live delta cells from preparations of human islets. This study identified LY6H as a novel cell surface marker of human pancreatic delta cells—a finding that will aid in the identification and characterisation of this important cell type.

**Supplementary Information:**

The online version contains supplementary material available at 10.1038/s41598-025-18321-2.

## Introduction

Previous studies have identified cell surface markers that are expressed on pancreatic endocrine cell types—including human pancreatic delta cells^1^. However, few of these markers are specific for individual endocrine cell types, and to date, no viable strategy has been shown for isolation of viable delta cells of the human pancreas.

Lymphocyte Antigen 6 Family Member H (LY6H) is a member of the LY6 family of proteins that belong to the Ly6/urokinase-type plasminogen activator receptor (uPAR) superfamily. This superfamily is characterised by the presence of a LU domain, a 60–80 amino acid region composed of 6–10 cysteine residues arranged to allow the formation of disulphide bridges which create a three-fingered (3 F) structural motif^2^. LY6H is believed to act as a modulator of nicotinic acetylcholine receptor (nAChR) activity^2^, and to play a role in glutamatergic signalling in the brain^3^. Several previous single-cell transcriptomic studies have identified *LY6H* transcripts in the delta cell population of human islets and pancreas^4,5^, however, analysis at the protein level has not been conducted.

In the present study, we show that LY6H is specifically expressed in delta cells of the adult human pancreas, in both control and diabetic subjects. Additionally, we demonstrate that antibodies against LY6H can be used to isolate viable human pancreatic delta cells for further downstream studies.

## Methods

### Ethical approval

Use of tissue donor material was approved by the St Vincent’s Hospital Human Research Ethics Committee (approval no. SVH HREC-A 011/04). All experiments were performed in accordance with relevant guidelines and regulations. Details of individual donors are provided in Tables 1 and 2.

**Table 1:** Information for donor material used in immunofluorescence studies.

	Unique identifier	Donor age (years)	Donor sex (M/F)	Donor BMI	Cause of death	Donor history of diabetes	Donor HbA1c	Diabetes duration (years)
Control-1	SVI-007-19	37	M	27.4	Hypoxic brain injury	No		
Control-2	SVI-009-19	52	M	26.5	Spontaneous intracranial haemorrhage	No		
Control-3	SVI-025-18	25	F	41.4	Cerebral hypoxia/ischemia	No		
T1D-1	SVI-004-18	46	M	29.0	Hypoxic brain injury	Yes, T1D	7.5	24
T1D-2	SVI-007-18	44	F	24.0	Cerebral infarction	Yes, T1D	11.4	11
T1D-3	SVI-012-19	59	F	28.7	Cerebral hypoxia/ ischemia	Yes, T1D	9	13
T2D-1	SVI-007-15	51	M	35.4	Brain dead	Yes, T2D	Unknown	Unknown
T2D-2	SVI-002-19	58	M	28.3	Brain dead	Yes, T2D	7.2	8
T2D-3	SVI-018-19	54	M	27.3	Brain dead	Yes, T2D	Unknown	Unknown

**Table 2:** Information for donor material used in flow sorting studies.

	Sort-1	Sort-2	Sort-3
Unique identifier	SVI-015-20	SVI-003-21	SVI-010-25
Donor age (years)	53	47	63
Donor sex (M/F)	F	M	F
Donor BMI	30.0	33.2	32.2
Cause of death	Intracranial haemorrhage	Intracranial haemorrhage	Cerebral infarction
Donor history of diabetes	No	No	No
Islet purity (%)	95%	85%	95%

### Islet isolation

Healthy human pancreata were obtained with informed consent from next of kin, for scientific purposes, from heart-beating, brain-dead donors, with research approval from the Human Research Ethics Committee at St Vincent’s Hospital, Melbourne. Human islets were purified by intraductal perfusion and digestion of the pancreases with collagenase AF-1 (SERVA/Nordmark, Germany)^6^ followed by purification using Ficoll density gradients^7^. Purified islets were cultured in Miami Media 1 A (Mediatech/Corning 98 − 021, USA) supplemented with 2.5% human serum albumin (Australian Red Cross, Melbourne, VIC, Australia), in a 37 °C, 5% CO_2_ incubator.

### HEK293T culture and transfection

Human embryonic kidney (HEK) 293T cells^8^ (ATCC CRL-11268) were maintained in Dulbecco’s modified Eagle’s medium (DMEM; Gibco; 10564011) supplemented with 10% fetal calf serum (FCS; Gibco; A4766801) and 1% GlutaMAX (Gibco; 35050061) and maintained at 5% CO_2_ at 37 °C. Cells were passaged upon reaching confluence using TrypLE Select (Life Technologies; 12563029). HEK293T cells were transfected with a pcDNA3.1(+)-P2A-eGFP (Addgene) plasmid containing the LY6H cDNA using Lipofectamine 3000 (Invitrogen; L3000015) as per the manufacturer’s directions. Cells were maintained in 5% CO2 at 37 °C for 48 h prior to immunofluorescence staining.

### Immunofluorescence staining

Paraffin sections of donor human pancreas were obtained from the Tom Mandel Islet Isolation Program (St Vincent’s Hospital, Victoria). These donor human pancreata were obtained with informed consent from next of kin, for scientific purposes, from heart-beating, brain-dead donors, with research approval from the Human Research Ethics Committee at St Vincent’s Hospital, Melbourne. Paraffin was removed using xylene, samples were rehydrated, and antigen retrieved using 10 mM citrate buffer. Samples were blocked for 1 h at room temperature in staining buffer (10% foetal calf serum (FCS) (Sigma-Aldrich; 12003 C) in PBS) and 0.1% Triton-X (Sigma-Aldrich; T9284), stained overnight with primary antibodies at 4 °C, stained for 1 h at room temperature with secondary antibodies, and stained with DAPI (Sigma-Aldrich; D9542) for 5 min. Antibody details are provided in Table 3. Samples were mounted using Fluoromount-G (Southern Biotech; 0100-01) and imaged using a LSM780 inverted confocal microscope (Zeiss). Image analysis was performed using ImageJ (version 1.0).

**Table 3:** Antibodies used for immunofluorescence and flow cytometry.

Antibody	Source	Catalogue number	Dilution	RRID
Mouse anti-LY6H	Abcam	ab55472	1:50	AB_2281371
Rabbit anti-SP-1 Chromogranin A	ImmunoStar	20,086	1:200	AB_572226
Rat anti-Somatostatin	Millipore	MAB354	1:200	AB_2255365
Guinea pig anti-INSULIN	Dako	A0564	1:400	AB_10013624
Rabbit anti-GLUCAGON	Dako	A0565	1:200	AB_10013726
Goat anti-guinea pig IgG (H + L) highly cross-adsorbed secondary antibody, Alexa Fluor 488	ThermoFisher	A-11,073	1:1500	AB_2534117
Goat anti-rabbit IgG (H + L), highly cross-adsorbed secondary antibody, Alexa Fluor 568	ThermoFisher	A-11,036	1:1500	AB_10563566
Goat anti-mouse IgG (H + L), cross-adsorbed secondary antibody, Alexa Fluor 647	ThermoFisher	A-21,236	1:1000	AB_2535805
Goat anti-rabbit IgG (H + L), cross-adsorbed secondary antibody, Alexa Fluor 488	ThermoFisher	A-11,008	1:1000	AB_143165
Goat anti-rat IgG (H + L), cross-adsorbed secondary antibody, Alexa Fluor 568	ThermoFisher	A-11,077	1:1000	AB_2534121

### Flow cytometry and sorting

Isolated human islets obtained from the Tom Mandel Islet Isolation Program were digested by resuspension in Accutase (Sigma-Aldrich; A6964) solution for 15 min at 37 °C. Following trituration, cells were washed in PBS and then stained with primary antibody in FACS buffer (2% FCS in PBS) for 30 min on ice. Cells were then washed twice with FACS buffer and stained with the appropriate secondary antibody for 30 min on ice. Antibody details are provided in Table 3. Cells were then washed twice with FACS buffer and then resuspended in 1 µg/ml propidium iodide (Sigma-Aldrich; P4864) to exclude dead cells, prior to cell sorting. Flow sorting was performed on a BD FACSAria Fusion (BD Biosciences). Data was collected and analysed using BD FACSDiva 8.0.1 (BD Biosciences). Isolated cells were resuspended in Trizol reagent (Sigma; 15596026) as a prelude to preparation of RNA.

### RNA extraction

Following isolation by flow sorting, cells were resuspended in TRIzol reagent (Thermo-Fisher; 15596026) and RNA extracted as per the manufacturer’s directions.

### Bulk RNAseq analysis

RNA extraction of FACS purified populations was performed using TRIzol extraction as directed by the manufacturer (Thermo-Fisher Scientific). RNA samples were processed, quality control performed, and sequenced by the Victorian Clinical Genetics Service, Melbourne (VCGS). Libraries were generated using an either a Truseq Stranded mRNA Library Prep Kit (Illumina) (Sort-1 donor) or a SMARTer Stranded Total RNA-seq Kit v3 - Pico Input Mammalian (Takara) (Sort-2 donor). Sequencing of samples was performed using an NovaSeq 6000 (Illumina) instrument. Between 20 and 30 million 150 bp paired-end reads were obtained per sample. Individual fastq files were aligned to the reference genome (GrCh38 assembly) with the Spliced Transcript Alignment to a Reference (STAR) software (version 2.7.3a)^9^ using default parameters. Nonuniquely mapping reads and read pairs with unpaired alignments were excluded. Read counts for each gene were determined using featureCounts as part of the Rsubread VERSION library. RNAseq analysis was performed on the raw count table using the limma^10^ and edgeR^11,12^ packages within R. Briefly, the counts per million (CPM) value was calculated using the cpm() function in edgeR, and genes expressed at low levels (defined as a CPM value below 0.5 in any sample) were filtered out. The filtered matrix was then used to create a DGEList object in edgeR, and the object TMM normalized using the calcNormFactors() function. For the Sort-2 donor, the count matrix was manually curated to remove sequences that were representative of non-coding RNA sequences. Highly variable genes were identified by estimating the variance of each gene across the different sort fractions from each donor, then sorting genes according to variance value. Unsupervised hierarchical clustering of samples was then performed using the top 1000 variable genes. The top 100 most variable genes for each donor is shown in Supplementary Figs. 1 and 2.

### Single cell RNAseq analysis

Processed RNA sequencing data was downloaded from GEO (GSE114412). The count matrix was filtered to remove mitochondrially-encoded genes, genes with less than 1000 UMIFM counts and cells with greater than 25% mitochondrial-DNA content. Variation in the total counts of individual cells was then removed by normalizing the sum of counts for each individual cell to 10,000. The normalized counts were then used for dimensionality reduction and clustering for each dataset, which was performed using the Seurat package within R^13^. Briefly, highly variable genes were identified using the FindVariableFeatures() function within Seurat. Principle components were then computed and clustering was performed using Louvain community detection in the space of the first 30 principle components. UMAP projections were then computed using the first 30 principle components. Differentially expressed genes within clusters were then computed using the FindAllMarkers() function within Seurat and cluster identity assigned using canonical genes as previously described^14^.

## Results

**Fig. 1:**
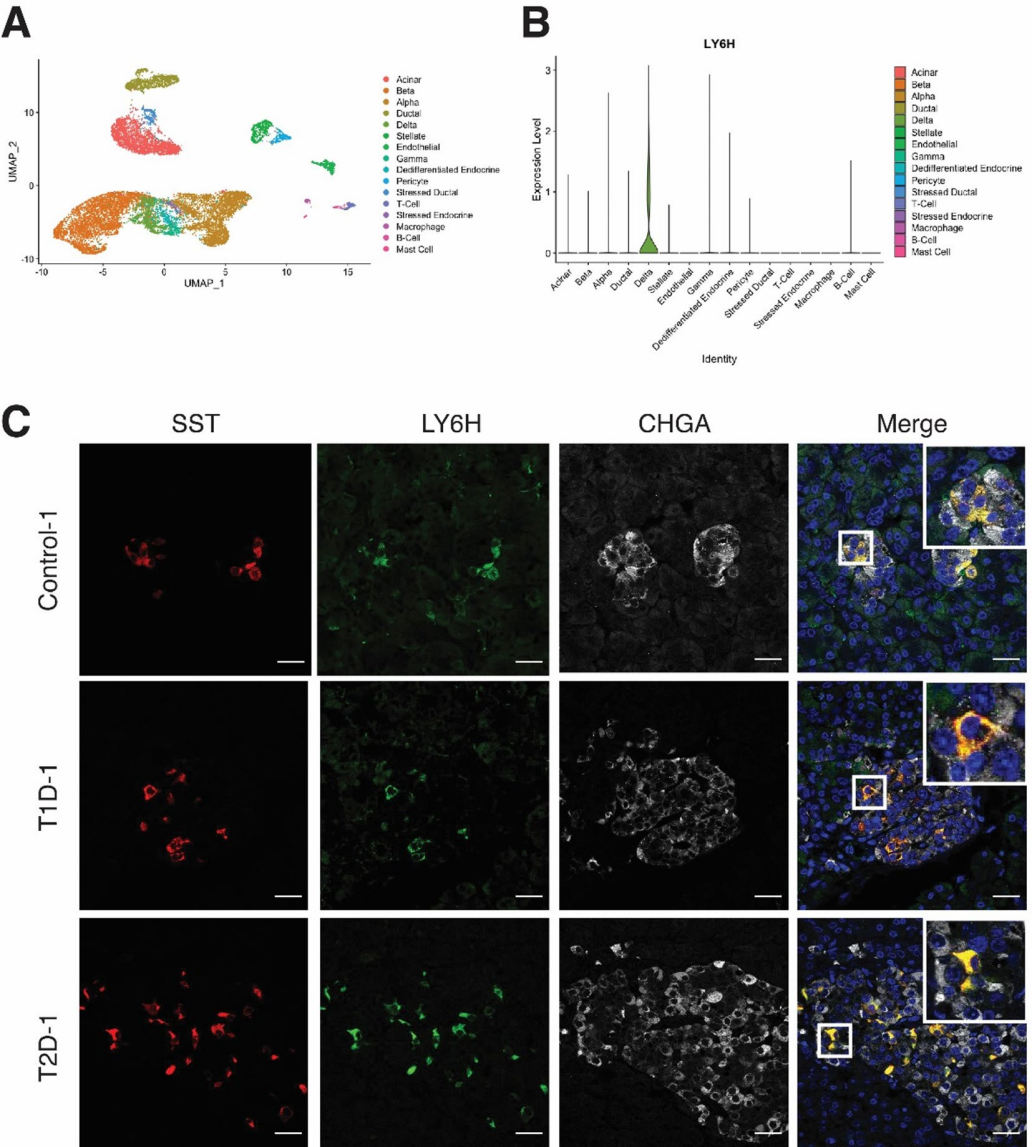
LY6H is a marker of human pancreatic delta cells. **(A)** Unsupervised clustering UMAP projection plot for 4 independent donors, with cell types as indicated. **(B)** Violin plot demonstrating expression of LY6H in the delta cells of the human pancreas as seen in scRNAseq analysis. **(C)** Immunofluorescence analysis of LY6H expression (green) in pancreatic sections representing control, T1D and T2D tissue donors, co-stained with antibodies recognising SOMATOSTATIN (SST, red) and CHROMOGRANIN A (CHGA, grey). Scale bars for all images are 25 μm.

We previously described the re-analysis of a single-cell RNA sequencing dataset (GSM3142001) with the view of identifying novel cell surface markers of specific pancreatic cell subsets^14^. Briefly, single-cell transcriptional profiling of islets from 4 independent non-diabetic donors were clustered and visualised using uniform manifold approximation and projection (UMAP) (Fig. 1a). Gene expression profiles associated with cell clusters were examined with a view to identifying cell surface markers that would be compatible with live cell analysis and purification techniques, such as flow cytometry. From this re-analysis, we identified *LY6H* transcripts as being specifically expressed in the delta cell cluster of this dataset (Fig. 1b). In order to examine this in a larger set of donors, a dataset from Elgamal et al.^15^ was analysed which contained 65 donors (11 autoantibody positive, 10 type 1 diabetic, 17 type 2 diabetic and 27 control). This analysis demonstrates that LY6H expression is found in the delta cell populations (Supplementary Fig. 3a–d). Whilst some cells are seen outside of the delta cell cluster in T1D and T2D donors to express LY6H, upon examination these cells also expressed SOMATOSTATIN (Supplementary Fig. 3e,f).

Consistent with the single-cell transcriptomic data, we found the LY6H expression was restricted to the delta cells within the islets, as identified by SOMATOSTATIN (SST) expression, with all SST-positive cells examined, seen to express LY6H (Fig. 1c). To emphasise this, we have reanalysed the images in Fig. 1 and Supplementary Fig. 4 to quantify the number of SST + cells that co-express LY6H. This analysis across more than 10 islets, demonstrated that all 129 SST-positive cells in these images expressed LY6H. The specificity of the LY6H antibody was validated using a transient expression system (Supplementary Fig. 5a). No co-expression of LY6H was seen within the beta (as marked by INSULIN) or alpha (as marked by GLUCAGON) cells in the islets (Supplementary Fig. 5b).

**Fig. 2:**
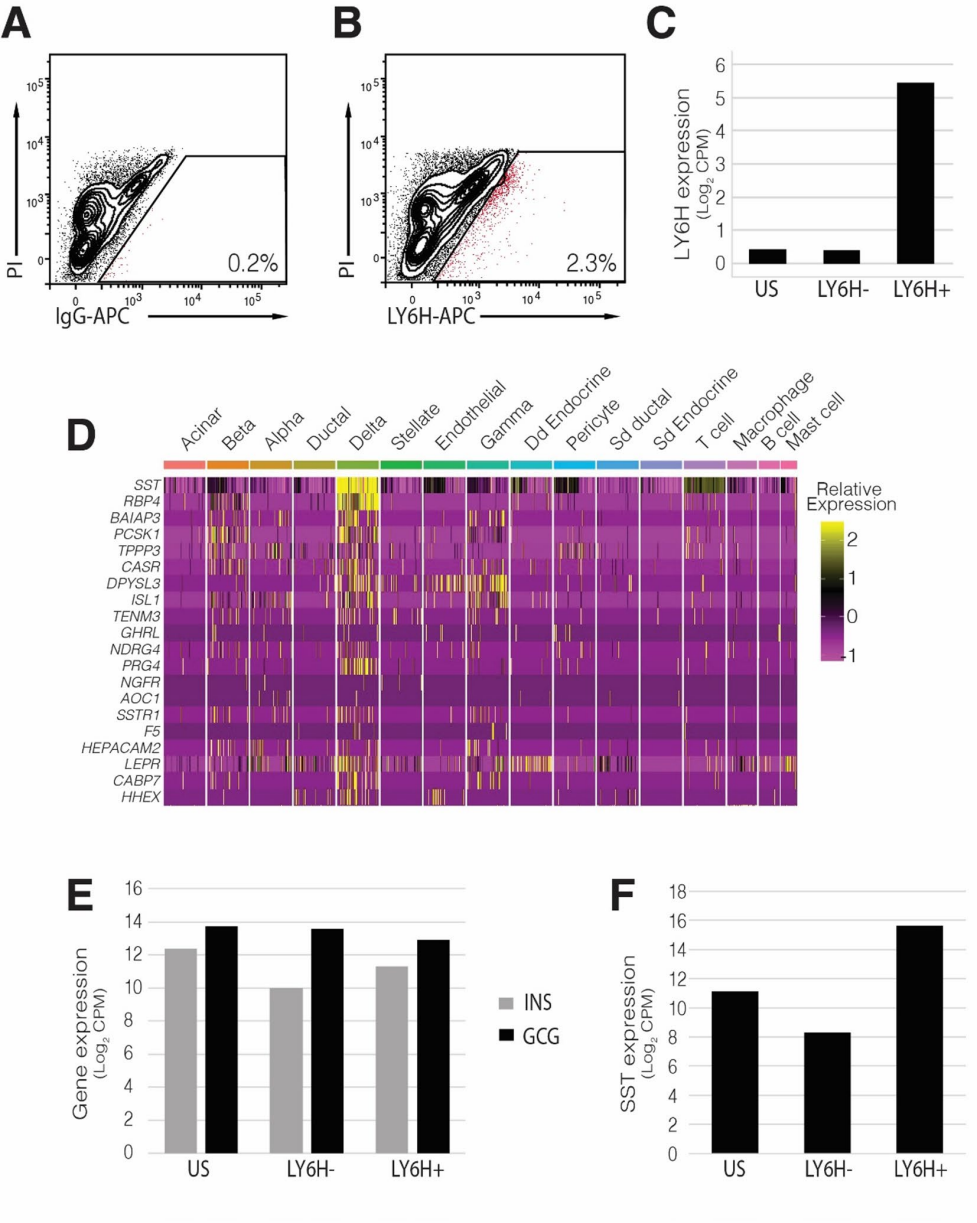
LY6H can be used to isolate human pancreatic delta cells. **(A)** Flow cytometry plot showing sort gates for human islets using a secondary (IgG-APC) antibody only (Sort-1 donor). **(B)** Flow cytometry plot showing sort gates for human islets stained an antibody directed against LY6H (Sort-1). **(C)** Transcript quantification (log2 counts per million (CPM) of LY6H expression in the indicated sorted fractions (US—unsorted). **(D)** Heatmap showing expression of the top 10 highly expressed genes in LY6H + cells from Sort-1 donor as identified by bulk RNA sequencing mapped against cell clusters from the scRNAseq analysis performed previously^6^. **(E)** Transcript quantification (log2 counts per million (CPM)) of INSULIN (INS) and GLUCAGON (GCG) expression in the indicated sorted fractions (US—unsorted). **(F)** Transcript quantification (log2 counts per million (CPM) of SOMATOSTATIN (SST) in the indicated sorted fractions (US—unsorted).

We next tested whether anti-LY6H antibodies could be used to isolate live delta cells from donor islets using flow cytometry. Flow cytometry analysis showed that only a small fraction of cells within the islet preparations expressed LY6H (Fig. 2a, b, Supplementary Fig. 5c), consistent with the relatively low abundance of delta cells within human islets. We isolated RNA from the cell LY6H + population, as well as from cells that did not express LY6H and from unsorted islet cells and subjected these samples to bulk RNAseq analysis (Fig. 2c, Supplementary Fig. 6).

To determine the identity of LY6H + cells, we mapped the 20 most highly expressed genes in this population to the gene expression profiles associated with specific cell clusters identified by scRNAseq analysis. This association, visualised by means of a heat map (Fig. 2d), demonstrates that LY6H is a specific marker of somatostatin expressing pancreatic delta cells. Additionally, it could be seen that while there was no enrichment in LY6H + cells for either INSULIN or GLUCAGON transcripts (Fig. 2e), it could be seen that there was an enrichment of SOMATOSTATIN transcripts in LY6H + cells (Fig. 2f) as compared to LY6H-negative cells or an unsorted cell fraction. Statistical analysis confirmed that there is a statistically significant increase in SOMATOSTATIN transcripts in the LY6H + cell fraction as compared to the unsorted cell fraction (*p* = 0.0114, *n* = 2). From an additional donor (SVI-010-25, Sort-3), we isolated both LY6H + and LY6H- cells and adhered these to poly-l-lysine coated coverslips. Cells were analysed by immunofluorescence for expression of SST. This analysis showed that the vast majority of cells isolated on the basis of LY6H expression also expressed SST, whilst cells that lacked LY6H expression also lacked SST expression (Supplementary Fig. 7).

These analyses confirm the results obtained from the whole pancreas immunofluorescence staining and verifies that LY6H is a novel cell surface marker that can be used to isolate delta cells from the human pancreas for downstream studies.

## Discussion

While previous reports have identified cell surface markers facilitating isolation of delta cells from the mouse pancreas^16^, this is the first report of a marker that enables the purification of a highly enriched population of viable delta cells from the human pancreas. This conclusion was based on flow cytometry experiments, which showed that anti-LY6H antibodies enabled isolation of a population of islet cells that was substantially enriched for somatostatin transcripts, a result consistent with our immunofluorescence analyses of human islets. In addition to enabling identification of delta cells in sections of control human pancreata, we found that LY6H also co-stained SST expressing delta cells in samples derived from both type 1 and type 2 diabetic donors. A limitation of our study is the relatively weak signal strength of the anti-LY6H antibody. This could be either due to the antibody affinity, or to the number of epitopes available for binding per cell. The issue of antibody affinity could be addressed by generating a new antibody with better signal to noise characteristics. Nevertheless, despite the relatively weak signal afforded by currently the available anti-LY6H antibody, our study demonstrates that LY6H can be used as a tool for the isolation of a highly enriched population of delta cells for downstream studies. In the future, further purification of this subset of endocrine cells may be aided by the identification of additional cell surface markers associated with either delta cells or other cell types present within islet cell populations.

LY6H has recently been identified as a modulator of α7 nAcetylcholine Receptor (α7 nAChR) function, where it’s extracellular domain interacts directly with α7 nAChR to inhibit ligand-induced channel activity^17^. In addition, this work demonstrated that soluble LY6H also possessed nAChR modulatory function, raising the possibility that LY6H activity may not be restricted to the cells which express it. It is well established that endocrine cells within the pancreatic islet are able to regulate each other’s function by both autocrine and paracrine signalling^18^. Previous studies in the mouse have identified expression of α7 nAChR in both the beta and alpha cells^19^. As LY6H is a GPI anchored protein, it is plausible that LY6H may be released from the surface of delta cells following the activation of phospholipases^20^ providing a potential mechanism by which it could affect neighbouring cells. In this scenario, secreted LY6H could modulate α7 nAChR expression on neighbouring alpha and/or beta cells, potentially affecting glucose homeostasis, independent of somatostatin secretion. In this context, it noteworthy that deletion of α7 nAChR in mice leads to a reduced beta cell mass, contributing to a predisposition for later weight gain^19^. These studies suggest that further investigation into the role of nAChR signalling in the endocrine pancreas function is warranted. In the same vein, it is well established that somatostatin secreted by delta cells modulates insulin and glucagon secretion^21^. In this light, the ability to purify human delta cells will allow for more detailed study of this cell type and any changes that occur during the diabetogenic process.

## Supplementary Information

Below is the link to the electronic supplementary material.

Supplementary Material 1

## Data Availability

Bulk RNA-seq data used in this study has been deposited in the Gene Expression Omnibus (GEO) data base and are available under the accessation number GSE264633. All other data supporting findings of this study are available from the corresponding author upon reasonable request.

## Abbreviations

T1D: Type 1 diabetes
