# Supplementary material for: LY6H is a marker of human pancreatic delta cells
Source: Sci Rep. 2025 Sep 26;15:33011. doi: 10.1038/s41598-025-18321-2 (PMC12474887; doi:10.1038/s41598-025-18321-2)
Supplement: Supplementary file 1 — Supplementary Material 1 [file 41598_2025_18321_MOESM1_ESM.pdf]

ESM

LY6H is a marker of human pancreatic delta cells

Jacqueline V. Schiesser[1,2,3,*], Yi Yu[1,2,3], Thomas Loudovaris[4], Helen E. Thomas[4,5], Andrew G. Elefanty[1,2,3,+], Edouard G. Stanley[1,2,3,+,*]

[1]Murdoch Children's Research Institute, The Royal Children's Hospital, Flemington Road, Parkville, Victoria 3052, Australia.

[2]The Novo Nordisk Center for Stem Cell Medicine (reNEW), Murdoch Children's Research Institute, Parkville, Victoria 3052, Australia

[3]Department of Paediatrics, Faculty of Medicine, Dentistry and Health Sciences, University of Melbourne, Parkville, Victoria 3052, Australia

[4]St. Vincent's Institute, Fitzroy, Victoria 3065, Australia

[5]Department of Medicine, St. Vincent's Hospital, University of Melbourne, Fitzroy, Victoria 3065, Australia

[+] Andrew G. Elefanty and Edouard G. Stanley contributed equally to this study.

*** Corresponding author**

Edouard G. Stanley

Murdoch Children's Research Institute, The Royal Children's Hospital, Flemington Road, Parkville, Victoria, 3052, Australia.

E-mail: ed.stanley@mcri.edu.au

Jacqueline V. Schiesser

Murdoch Children's Research Institute, The Royal Children's Hospital, Flemington Road, Parkville, Victoria, 3052, Australia.

E-mail: jacqui.schiesser@mcri.edu.au

Keywords Delta cell • Type 1 diabetes • Cell surface markers • Islet • Cell isolation

Abbreviations T1D, Type 1 diabetes

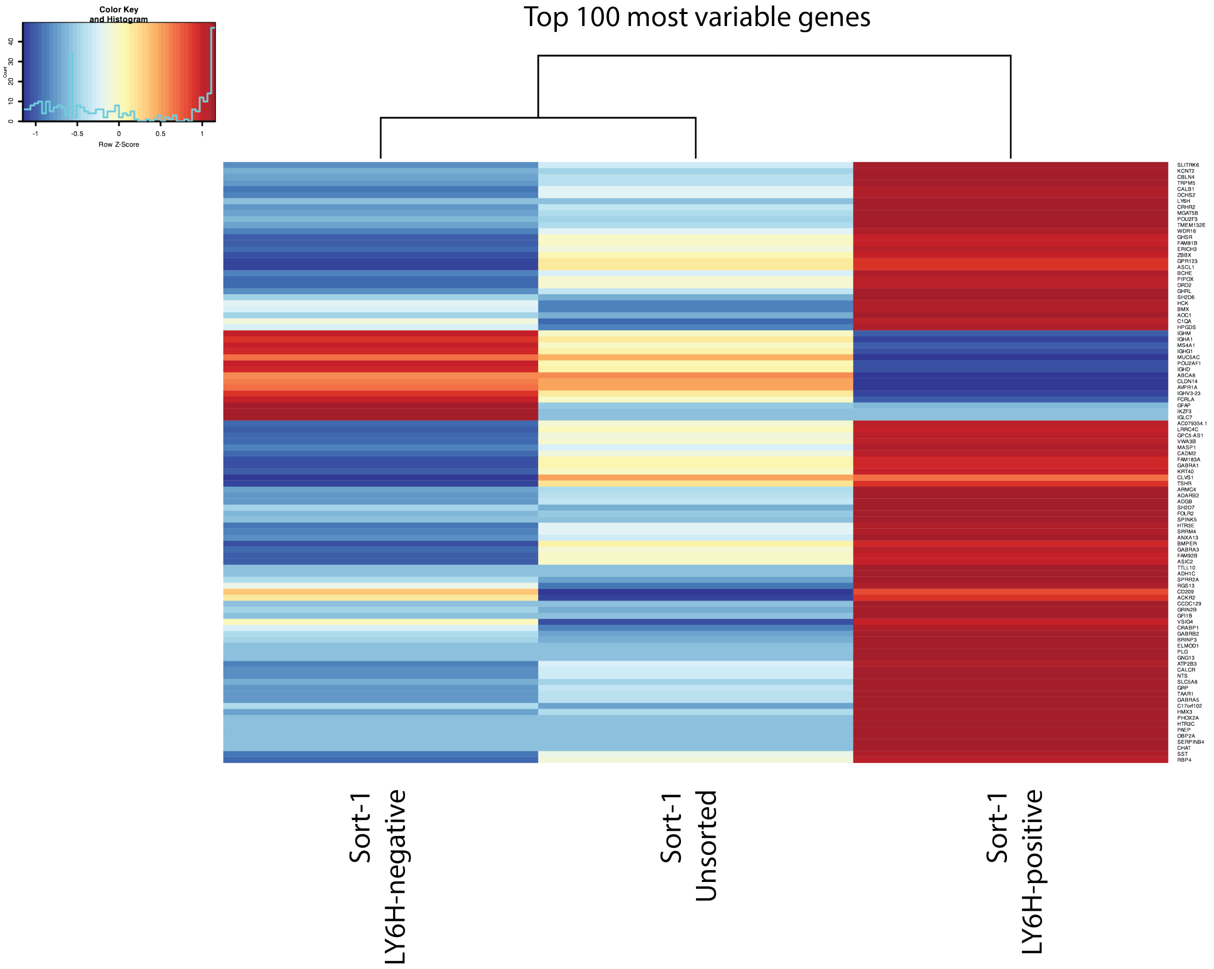

Supplementary Figure 1. RNA-seq analysis of populations isolated using antibodies against LY6H from a human islet preparation. Heatmap of the top 100 variable genes in samples sorted from donor Sort-1. The heatmap was generated using the heatmap2() function within the gplots package (version 3.1.1) in R.

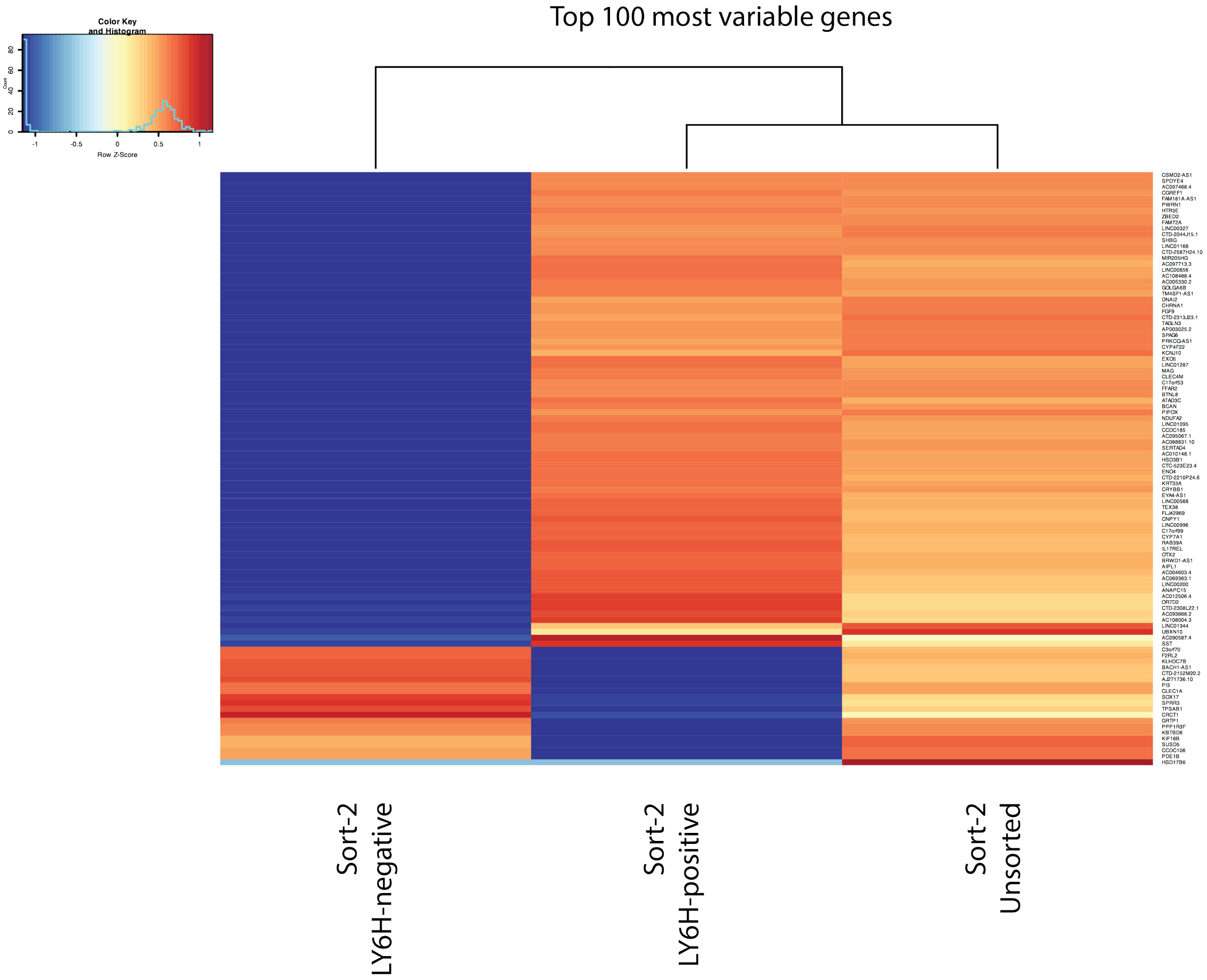

Supplementary Figure 2. RNA-seq analysis of populations isolated using antibodies against LY6H from a human islet preparation. Heatmap of the top 100 variable genes in samples sorted from donor Sort-2. The heatmap was generated using the heatmap2() function within the gplots package (version 3.1.1) in R.

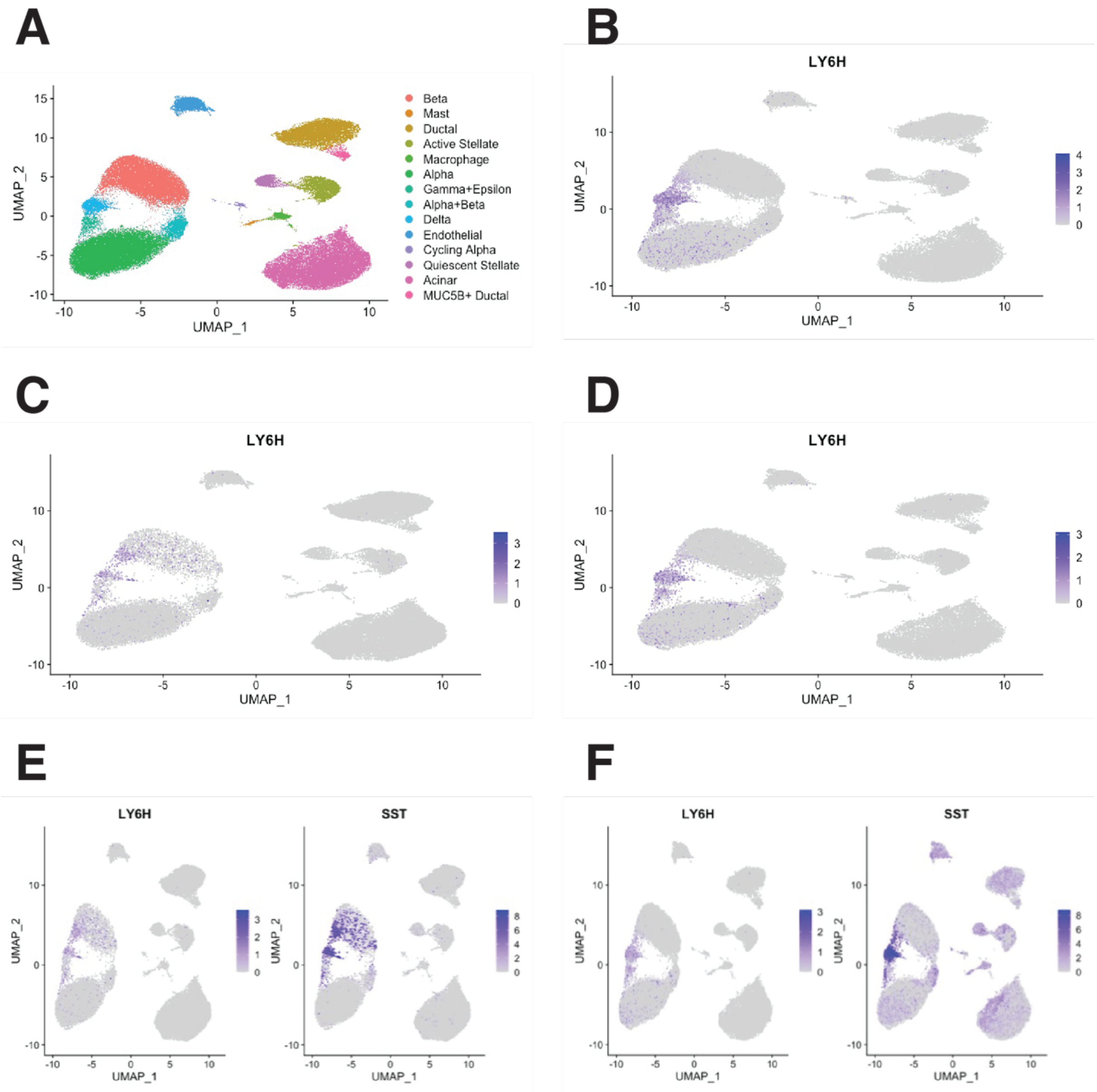

Supplementary Figure 3. scRNAseq analysis demonstrates that LY6H is a marker of human pancreatic delta cells. (A) Unsupervised clustering UMAP projection plot for 65 independent donors, with cell types as indicated. **(B)** Feature plot demonstrating expression of LY6H in the delta cells of the human pancreas from control donors. **(C)** Feature plot demonstrating expression of LY6H in the delta cells of the human pancreas from donors with T1D. **(D)** Feature plot demonstrating expression of LY6H in the delta cells of the human pancreas from donors with T2D. **(E)** Feature plot demonstrating the expression of LY6H and SST in the pancreas of donors with T1D. **(F)** Feature plot demonstrating the expression of LY6H and SST in the pancreas of donors with T2D.

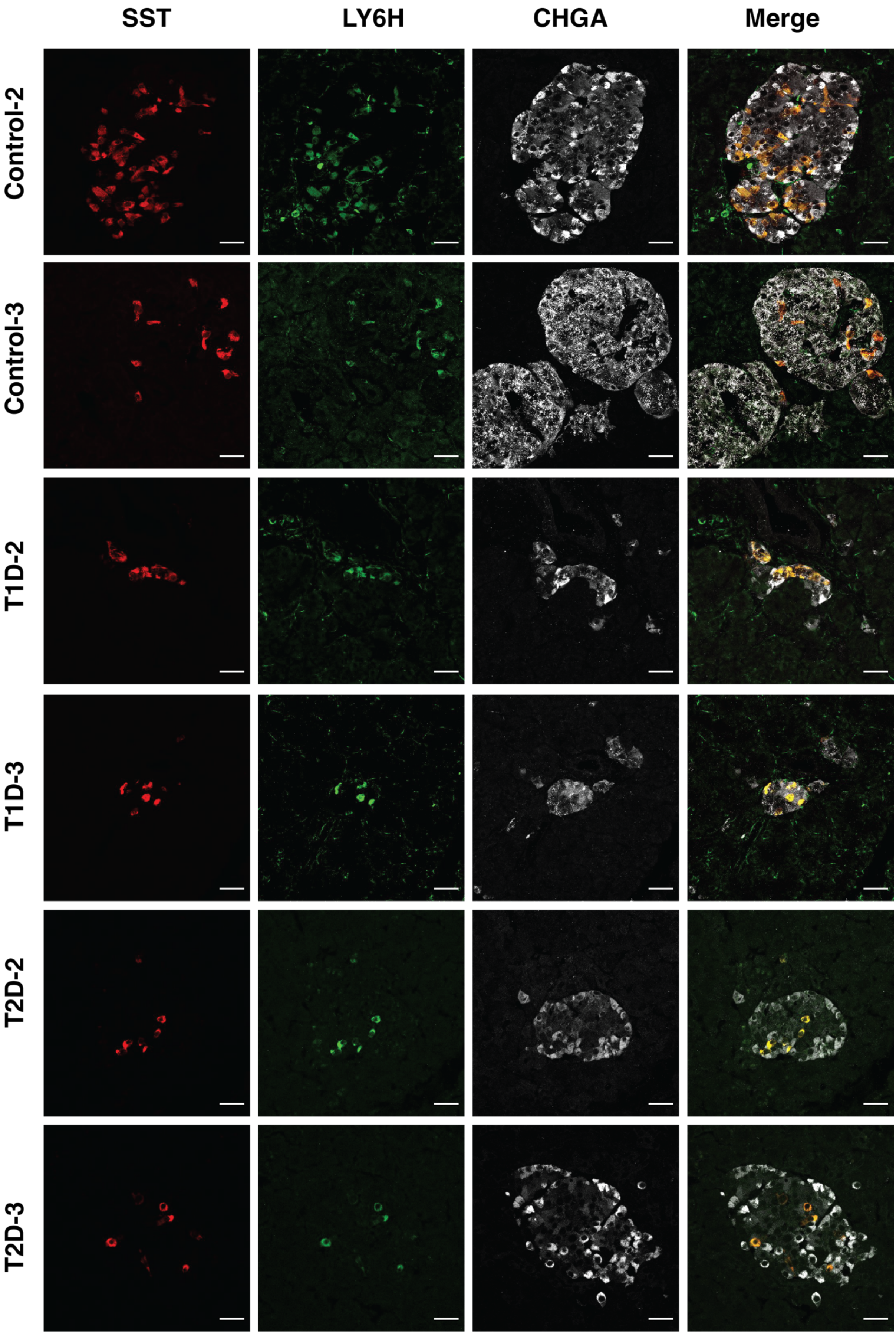
SST
LY6H
CHGA
Merge
Control-2
Control-3
T1D-2
T1D-3
T2D-2
T2D-3

Supplementary Figure 4. Expression of LY6H in human pancreas from additional control and diabetic pancreata to those analysed in the main figures.

(A) Immunofluorescence analysis of LY6H (green) expression in sections of pancreatic tissue derived from additional control, T1D and T2D tissue donors, co-stained with antibodies recognising SOMATOSTATIN (red) and CHROMAOGRANIN A (grey). Scale bars for all images are 25μm.

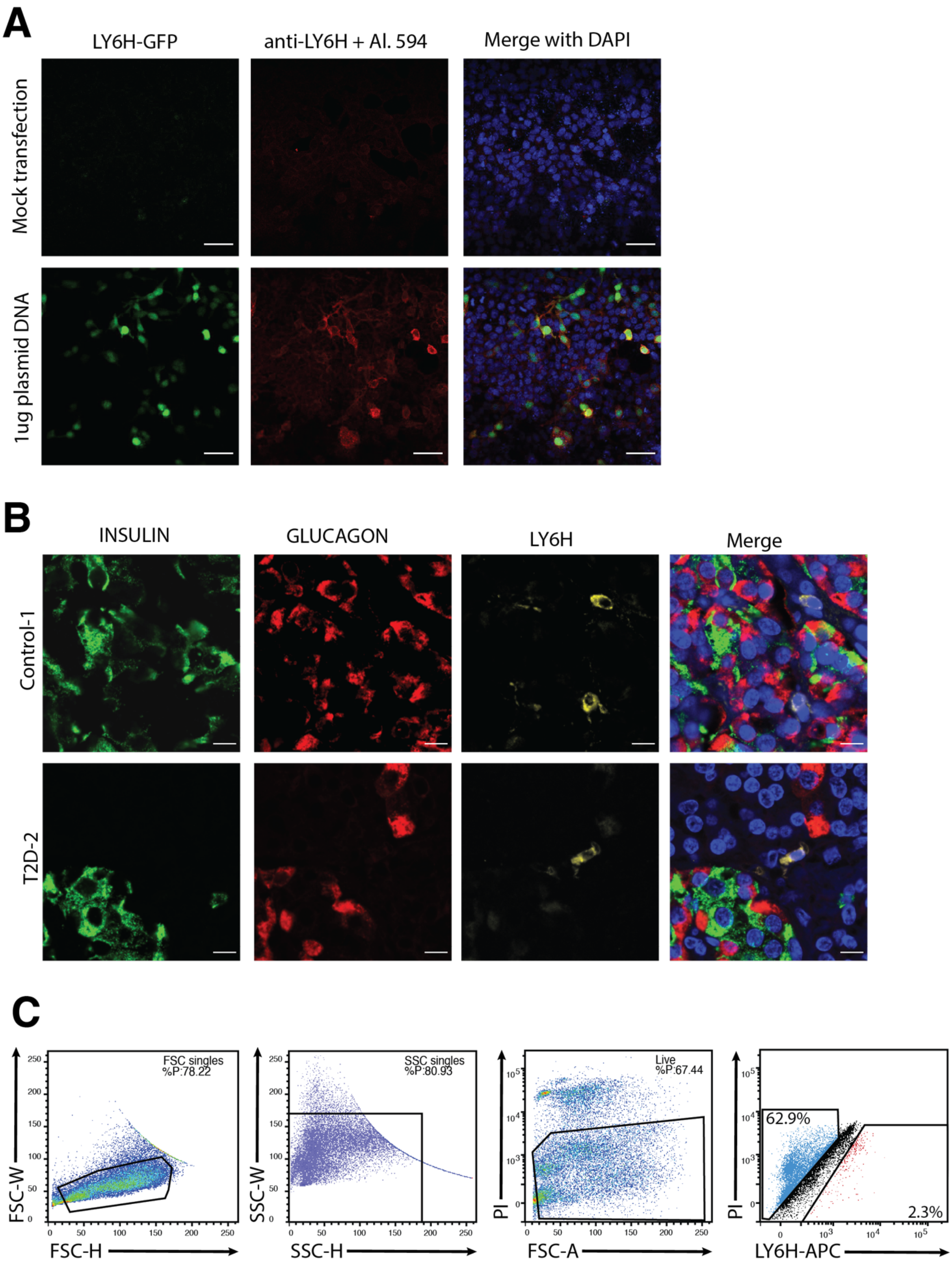

Supplementary Figure 5. LY6H antibodies identify delta cells in the human islet. (A) 293T cells either mock transfected or transfected with a plasmid containing LY6H cDNA and GFP were labelled with a LY6H antibody 48 hours post-transfection. It can be seen that only LY6H-GFP+ transfected cells are labelled using a LY6H antibody, demonstrating the specificity of the LY6H antibody. **(B)**

Immunofluorescence analysis of LY6H (yellow) expression in sections of pancreatic tissue derived from control donor-1 or type 2 diabetic (T2D) donor-2, co-stained with antibodies recognising GLUCAGON (red) and INSULIN (green). Scale bars for all images are 10μm. **(C)** Flow cytometry sort gating strategy for the isolation of LY6H+ and LY6H- fractions from sort donor-1.

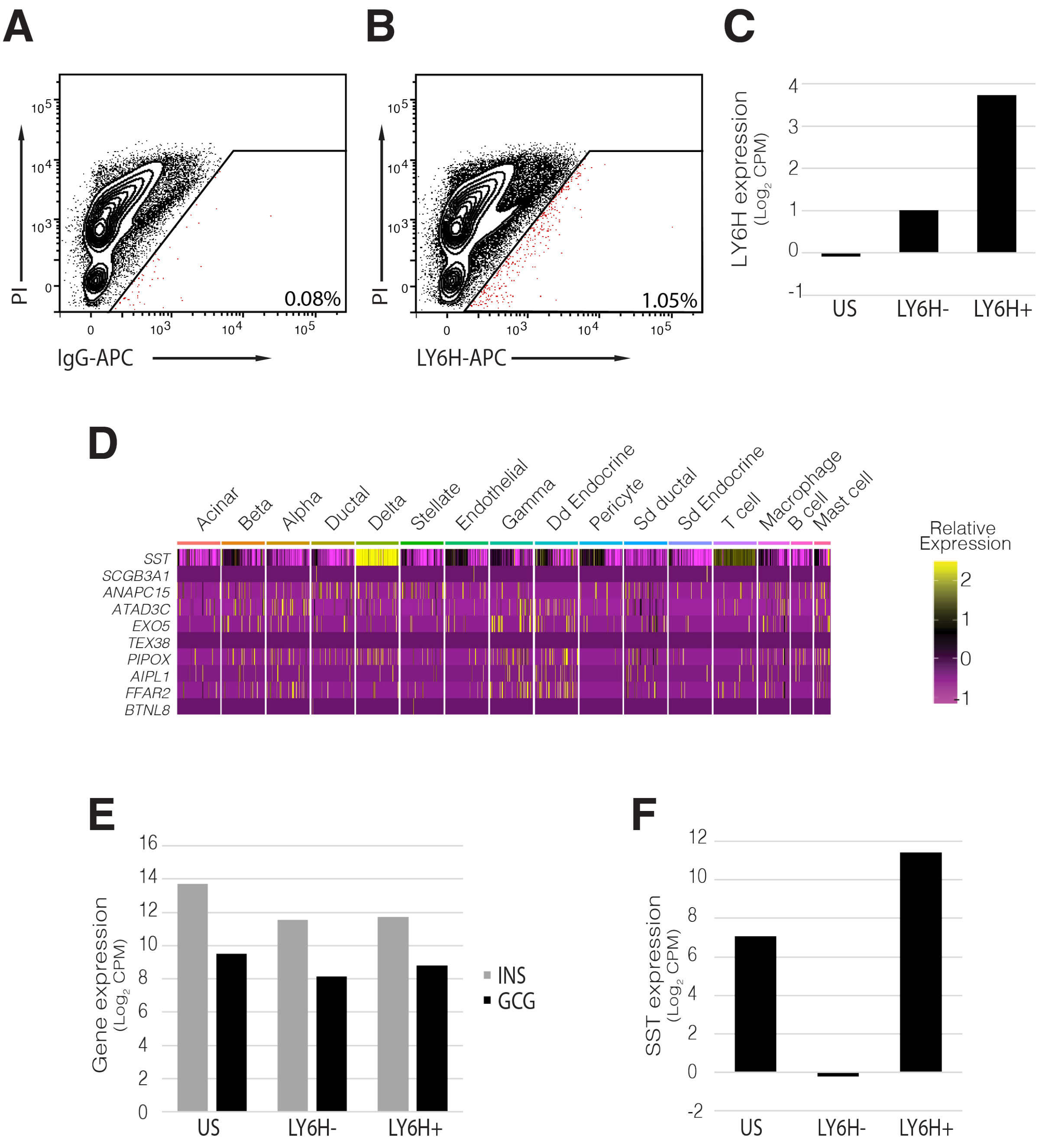

Supplementary Figure 6. Expression of LY6H transcripts from an additional control donor

A) Flow cytometry plot showing sort gates for human islets using a secondary (IgG-APC) antibody only (Sort-2 donor) **(B)** Flow cytometry plots showing sort gates for human islets stained with an antibody directed against LY6H (Sort-2 donor) **(C)** Transcript quantification (log2 counts per million (CPM)) of LY6H expression in the

indicated sorted fractions (US – unsorted). **(D)** Heatmap showing expression of the top 10 highly expressed genes in LY6H+ cells from Sort-2 donor as identified by bulk RNA sequencing mapped against cell clusters from the scRNAseq analysis performed previously[6]. **(E)** Transcript quantification (log2 counts per million (CPM)) of INSULIN (INS) and GLUCAGON (GCG) expression in the indicated sorted fractions (US – unsorted). **(F)** Transcript quantification (log2 counts per million (CPM) of SOMATOSTATIN (SST) in the indicated sorted fractions (US – unsorted).

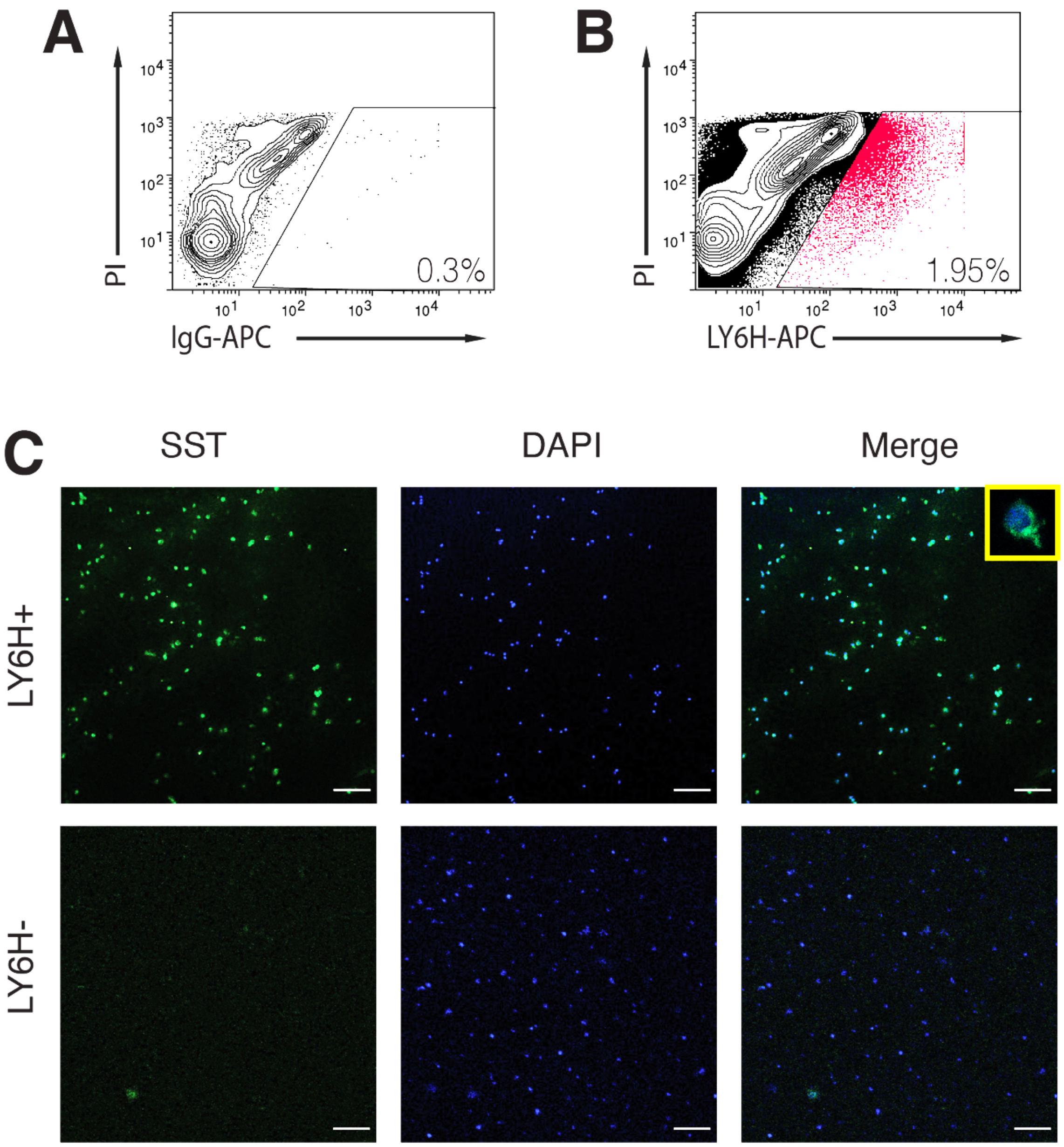

Supplementary Figure 7. Expression of SOMATSTATIN in sorted LY6H fractions from an additional control donor

A) Flow cytometry plot showing sort gates for human islets using a secondary (IgG-APC) antibody only (Sort-3 donor) **(B)** Flow cytometry plots showing sort gates for human islets stained with an antibody directed against LY6H (Sort-3 donor) **(C)** SOMATOSTATIN expression in sorted LY6H+ and LY6H- cells
